# Association between immune cells and multiple cancers: Insights from Mendelian randomization and gene-based analysis

**DOI:** 10.1016/j.isci.2025.114618

**Published:** 2026-01-02

**Authors:** Mingshuang Tang, Huijie Cui, Xueyao Wu, Yutong Wang, Xunying Zhao, Rong Xiang, Jinyu Xiao, Lin Chen, Yanqiu Zou, Yunjie Liu, BoWen Lei, Xiaofeng Ma, Di Zhang, Mengyu Fan, Jiayuan Li, Xia Jiang, Ben Zhang

**Affiliations:** 1 Department of Epidemiology and Biostatistics, West China School of Public Health and West China Fourth Hospital, Sichuan University, Chengdu, China; 2 Department of Epidemiology and Health Statistics, School of Public Health, Southwest Medical University, Luzhou, Sichuan, China; 3 Department of Nutrition and Food Hygiene, West China School of Public Health and West China Fourth Hospital, Sichuan University, Chengdu, China; 4 Department of Clinical Neuroscience, Karolinska Institute, Stockholm, Sweden; 5 Department of Occupational and Environmental Health, West China School of Public Health and West China Fourth Hospital, Sichuan University, Chengdu, China; 6 Departments of Cardiology, Neurology, and Oncology, Hainan General Hospital and Hainan Affiliated Hospital, Hainan Medical University, Haikou 570311, China

**Keywords:** Health sciences

## Abstract

To investigate the roles of immune cells in cancer, we conducted Mendelian randomization (MR) analyses of 731 immune cells and 16 site-specific cancers, alongside gene-based and enrichment analyses. MR identified 79 significant associations (46 protective, 33 risk-enhancing). Key findings include BAFF-R on B cells lowering melanoma risk, and CD27 on B cells increasing lung cancer risk. Associations were also observed with breast, ovarian, cervical, and other cancers, but no significant association was found with oral cavity or pancreatic cancers. Gene-based analysis revealed 61 shared genes between immune cells and cancers. 56 genes were associated with prostate cancer, 30 with lung cancer, three with breast cancer, two with cervical cancer, and one with melanoma. Interestingly, several prostate cancer-associated genes were also associated with breast, cervical, and lung cancers. These findings suggest potential biological mechanisms linking immune cells to cancer development and progression, highlighting the complex role of the immune system in cancer.

## Highlights

• Genome-wide assessment of pleiotropy between 731 immunophenotypes and 16 site-specific cancers
• MR identified 79 significant causal associations between immunophenotypes and cancer risk
• Gene-based analysis revealed 61 shared genes between immunophenotypes and cancers

## Graphical abstract

**Figure:**
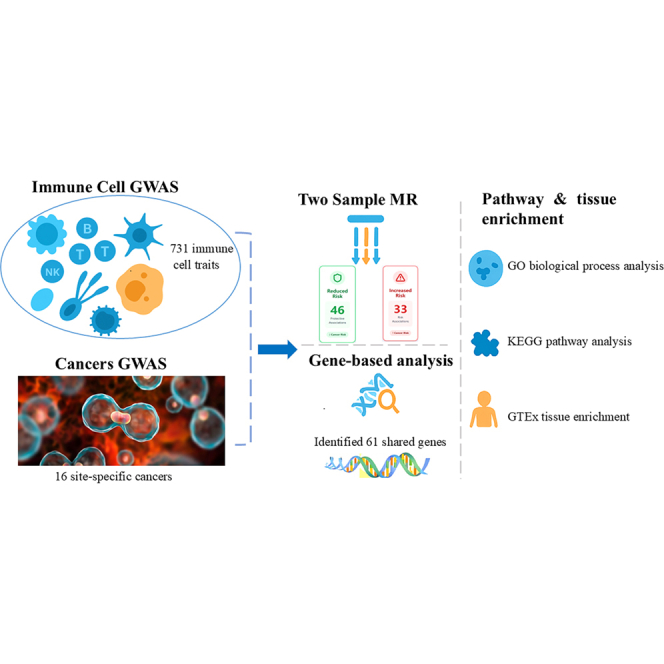

## Introduction

Immune cells are essential in both the development and progression of cancer.^1^^,^^2^ Traditional understanding posits that intact immune responses—such as immune surveillance or immunoediting—are essential for preventing and inhibiting cancer development.^3^^,^^4^ Conversely, accumulating evidence has elucidated specific mechanisms of immune regulation in carcinogenesis, such as pro-tumorigenic inflammation, immune surveillance suppression mediated by TH17 and regulatory T cells (Tregs), TH1-mediated immunosuppression, and local and distant tumorigenesis regulated by microbiota via alterations in the inflammatory and metabolic pathways.^5^^,^^6^^,^^7^^,^^8^ Additionally, clinical and preclinical studies have indicated the crucial contribution of peripheral immune cells to an anti-tumor immune response.^9^ Overall, these studies highlight the influence of the immune system on alterations in cancer cells, potentially paving the way for enhanced strategies in cancer prevention.

Epidemiological studies have linked the immune system with cancer. A previous study involving a total of two million cases of 101 cancers highlighted the significant impact of a declining immune system, particularly reduced T cell output, on cancer development.^10^ Additionally, a population-based cohort study investigated the association between four immune-related markers based on blood cell counts: neutrophil-to-lymphocyte ratio (NLR), platelet-to-lymphocyte ratio (PLR), and systemic immune-inflammatory index (SII) with cancer risk, revealing that elevated SII serves as a robust and independent risk indicator for developing multiple cancers.^11^ Another observational study using UK Biobank data identified positive associations between SII, NLR, PLR, and risk for seven out of 17 cancers.^12^ Despite these insights, existing research has predominantly focused on certain immune markers (e.g., SII), failing to explore the unique contributions of various immune cells to site-specific cancer risk. Therefore, there is a need for detailed investigations into the specific roles of various immune cells in the pathogenesis of cancer, to serve as a reference for precise guidance on cancer prevention and treatment.

As phenotypic correlations derived from observational designs can be subject to bias, confounding, and reverse causality, Mendelian randomization (MR) analysis offers more robust insights into the causal associations between risk factors and disease, most closely resembling a randomized controlled trial (RCT).^13^ Published MR analyses on the relationship between immune cells and cancer risk have covered several cancers including breast,^14^^,^^15^ ovarian,^15^ endometrial,^15^ lung,^16^^,^^17^ and gastrointestinal tract cancers.^18^ Additionally, inconsistencies in MR methods across these studies have made it challenging to compare the robustness of associations. Therefore, a comprehensive study to assess the relationship between various immune cells and multiple site-specific cancers using a systematic approach is needed. Moreover, investigations into the shared genetic basis and underlying genetic mechanisms linking immune cells and cancer remain unexplored.

**Figure 1:**
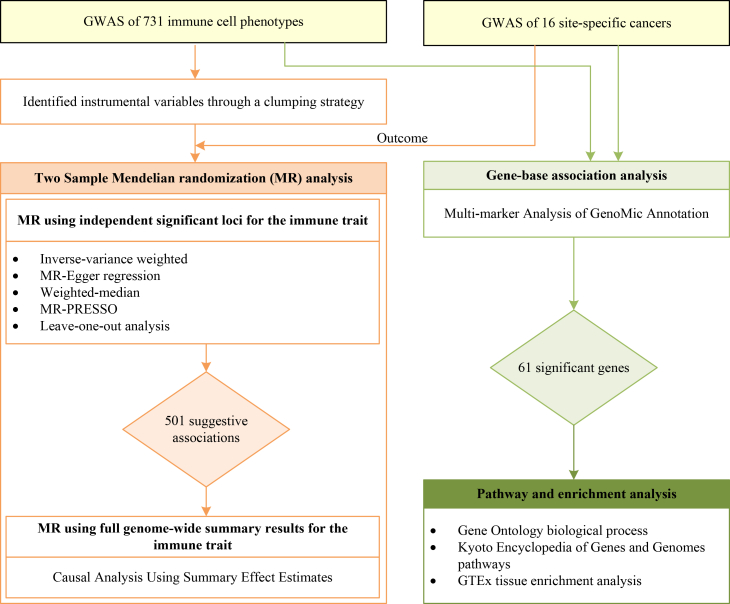
A schematic framework for the overall design of this study. PRESSO, Pleiotropy RESidual Sum and Outlier

To sum up, in this study, we aimed to investigate the relationship between 731 genetically predicted immune cell phenotypes and 16 site-specific cancers with available genome-wide association studies (GWASs) data, by conducting MR analysis, followed by gene-based analysis, as well as pathway and tissue enrichment analyses. Our aim was to enhance the understanding of the intricate interactions between immune cell biology and cancer susceptibility, potentially revealing novel insights to inform future preventive and therapeutic strategies. The overall study design is illustrated in Figure 1.

## Results

### Mendelian randomization analysis

**Figure 2:**
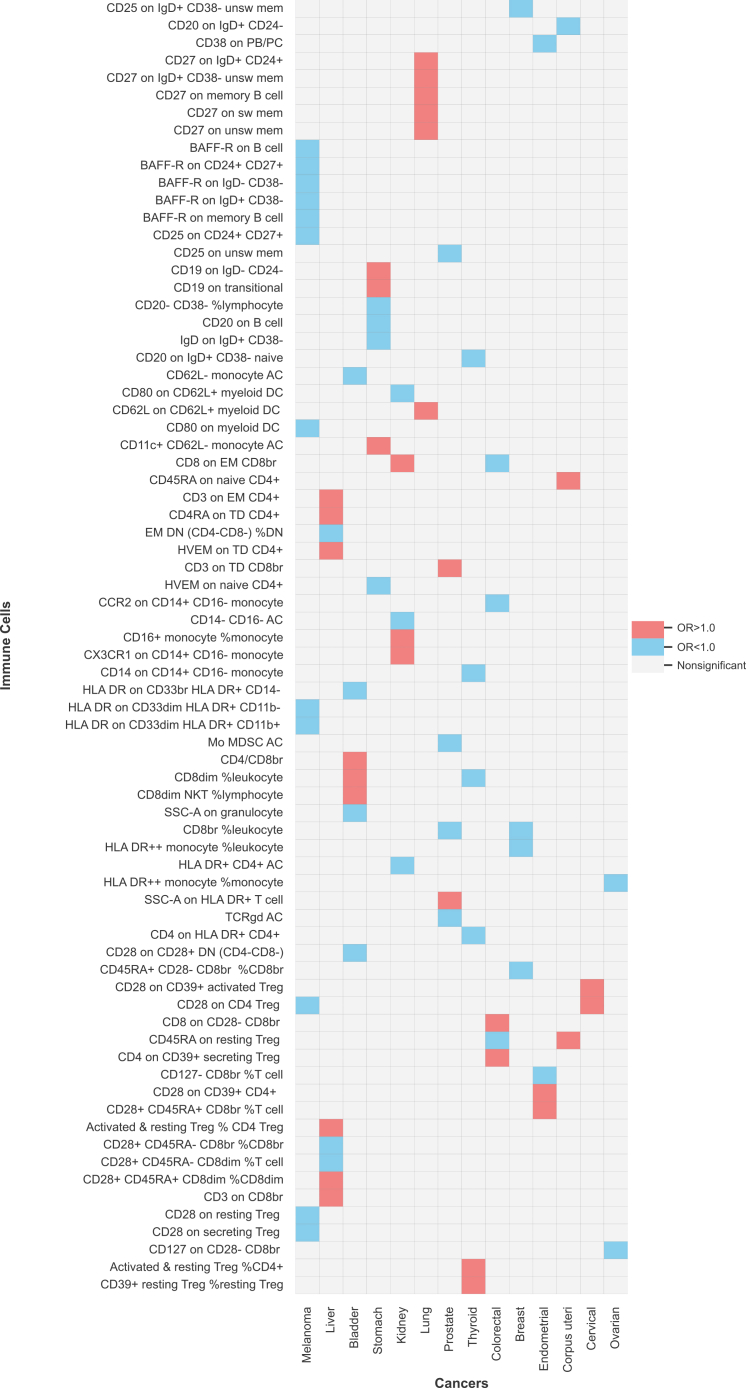
The significant results of Mendelian randomization analysis for causal associations between immune cells and cancer risk The red squares represent positive associations, the blue squares represent negative associations, and the gray squares represent non-significant associations. OR indicates odds ratio.

In MR analyses, we identified 506 suggestive causal associations using independent significant loci associated with immune cells, where the P-value was <0.05 in IVW, and consistent effect size direction across all complementary methods, including MR-Egger regression, weighted median, and MR-PRESSO (Data S1). However, the leave-one-out analysis results revealed that the causal estimates of five associations were affected by outliers (Table S1 and Figure S1). Consequently, 501 associations were included in the MR CAUSE analysis (using genome-wide summary results associated with immune cells) Table S2), of which 79 were found to be statistically significant (Table 1; Figure 2; Table S3).

**Table 1:** Summary of the number of exposures (immune cells) associated with each outcome (cancer) in Mendelian randomization analyses

Outcome	Excluded exposures		P ≥0.05 in IVW	*p* < 0.05 in IVW		Significant associations	Total exposures
	NO. of SNP <4	F-statistics <10		Inconsistence in sensitivity analyses	Inconsistence in MR-CAUSE		
Bladder cancer	0	13	687	4	20	7	731
Breast cancer	0	22	672	4	29	4	731
Cervical cancer	53	0	651	10	15	2	731
Colorectal cancer	0	18	682	1	25	5	731
Corpus uteri cancer	0	10	686	1	31	3	731
Endometrial cancer	0	7	690	2	28	4	731
Kidney cancer	3	0	686	12	24	6	731
Liver cancer	1	3	659	1	58	9	731
Lung cancer	0	7	694	1	23	6	731
Melanoma	0	13	673	2	31	12	731
Oral cavity cancer	1	4	703	1	22	0	731
Ovarian cancer	0	24	667	3	35	2	731
Pancreatic cancer	684	0	42	1	4	0	731
Prostate cancer	0	24	669	3	29	6	731
Stomach cancer	0	10	688	1	25	7	731
Thyroid cancer	0	10	691	1	23	6	731

IVW, inverse variance weighted; MR, Mendelian randomization; CAUSE, causal analysis using summary effect estimates.

Among the 79 significant associations involving 74 immune cell phenotypes and 14 site-specific cancers, 46 indicated that immune cells were associated with a reduced risk of cancer, while 33 suggested an increased risk. Notably, we identified 12 distinct immune cells significantly associated with a reduced risk of melanoma. These include BAFF-R expression on five B cell types, CD25 on CD24^+^ CD27^+^ B cell, CD80 on myeloid DC, CD28 on CD4^+^ Treg cell, CD28 on resting Treg cell, CD28 on secreting Treg cell, HLA DR on CD33dim HLA DR + CD11b-myeloid cell, and HLA DR on CD33dim HLA DR + CD11b+ myeloid cell. Additionally, we found four immune cells associated with a reduced risk of breast cancer: CD25 on IgD+ CD38^−^unswitched memory B cell, CD45RA + CD28^−^ CD8br %CD8br Treg cell, CD8br T cell %leukocyte, and HLA DR++ monocyte %leukocyte. Furthermore, two immune cells were associated with a reduced risk of ovarian cancer: CD127 on CD28^−^ CD8br Treg cell and HLA DR++ monocyte %monocyte. In contrast, six immune cells were associated with an increased risk of lung cancer: CD27 expression on five B cell types, and CD62L on CD62L + myeloid DC. Additionally, two immune cells were associated with an increased risk of cervical cancer: CD28 on CD39^+^ activated Treg cells and CD28 on CD4 Treg cells.

However, among the remaining nine cancers, distinct immune cells showed varying associations with cancer risks. Specifically, three immune cells were associated with a decreased risk of liver cancer, whereas six indicated an increased risk. Four immune cells were associated with a decreased risk of bladder cancer, while three were associated with an increased risk. Similarly, four immune cells were associated with a decreased risk of stomach cancer, alongside three that were associated with an increased risk. In the case of kidney cancer, three immune cells were associated with a lower risk, while three indicated a higher risk. For prostate cancer, four immune cells were associated with a reduced risk, whereas two indicated an elevated risk. Furthermore, four immune cells showed a decreased risk of thyroid cancer, while two were associated with an increased risk. Additionally, three immune cells showed a decreased risk of colorectal cancer, whereas two were associated with an increased risk. We also identified two immune cells associated with a decreased risk of endometrial cancer, alongside two that increased it. Finally, one immune cell was associated with a decreased risk of corpus uteri cancer, while two immune cells were associated with an increased risk.

Besides, we found no significant association between immune phenotypes and oral cavity or pancreatic cancers.

### Gene-based analysis

**Figure 3:**
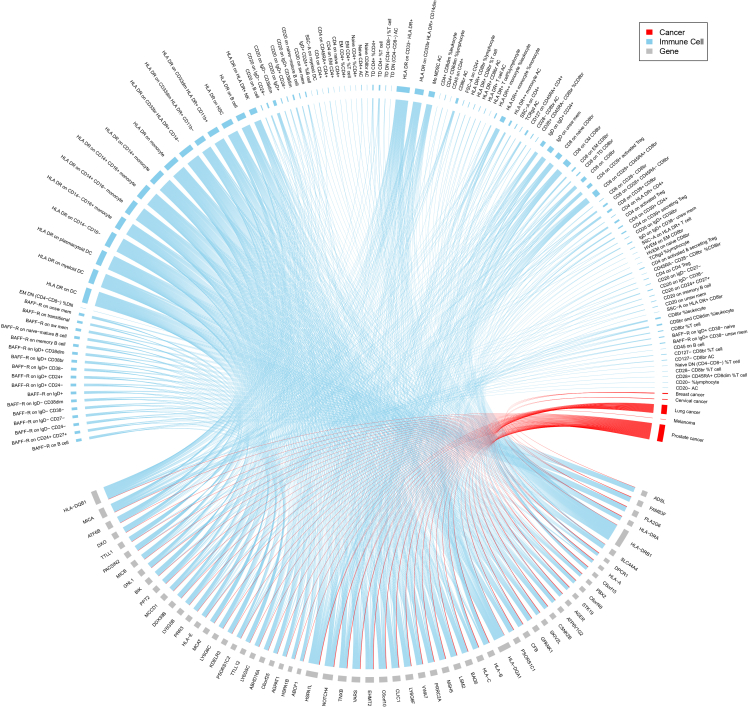
A chord plot indicating the genes that significantly associate with immune cells and cancers Genes are located on the bottom half of the plot, while immune cells and cancer types are on the top half. Significant associations (*p* < 2.57 × 10^−6^) are shown as solid lines. Blue lines indicate significant associations with immune cells. Red lines indicate significant associations with cancers.

Through MAGMA, we selected 61 genes that showed significance in at least 10 out of 731 immune cell phenotypes. Subsequently, we performed an intersection analysis to assess the overlaps between these 61 genes and those significantly associated with each cancer, ultimately encompassing a total of 121 immune cells and five cancers (Figure 3; Table S4).

Among these shared associations, 56 genes were shared between 103 immune cells and prostate cancer. Specifically, 49 of these genes are located on chromosome 6 (p22.1-p21.32) and associated with seven immune cell types (including B cells characterized by CD20 and IgD expression, Treg cells by CD4, CD8, CD28, and CD127 expression, TNBK type by CD4, CD8, and HLA-DR expression, T cell type by CD8 and TD expression, cDC type by HLA-DR expression, myeloid cell types by Mo MDSC AC and HLA-DR expression, and monocyte types by HLA-DR expression). The remaining seven genes are located on chromosome 22 (q13.1-q13.2) and are associated with BAFF-R expression on B cell type. Additionally, 30 genes located within the Human leukocyte antigen (HLA) region on chromosome 6 were shared between 75 immune cells (including B cells characterized by CD20 and IgD expression, Treg cells by CD4, CD8, and CD127 expression, TNBK type by CD4, CD8, and HLA-DR expression, T cell type by CD8 and TD expression, cDC type by HLA-DR expression, myeloid cell type by Mo MDSC AC and HLA-DR expression, and monocyte type by HLA-DR expression) and lung cancer. Furthermore, three genes on chromosome 22q13.1 were shared between 18 immune cells belonging to the B cell type characterized by BAFF-R expression and breast cancer. Moreover, two genes within the HLA region were associated with 59 immune cells (including B cells characterized by CD20 and IgD expression, Treg cells by CD28 and CD127 expression, TNBK subsets by CD4 and HLA-DR expression, T cell subsets by CD8 and TD expression, cDC subsets by HLA-DR expression, myeloid cell subsets by Mo MDSC AC and HLA-DR expression, and monocyte subsets by HLA-DR expression) and cervical cancer. One gene was shared between 18 immune phenotypes belonging to B cells characterized by BAFF-R expression and melanoma. Interestingly, several genes associated with prostate cancer were also linked to breast, cervical, and lung cancers, respectively.

### Pathway and tissue enrichment analysis

**Figure 4:**
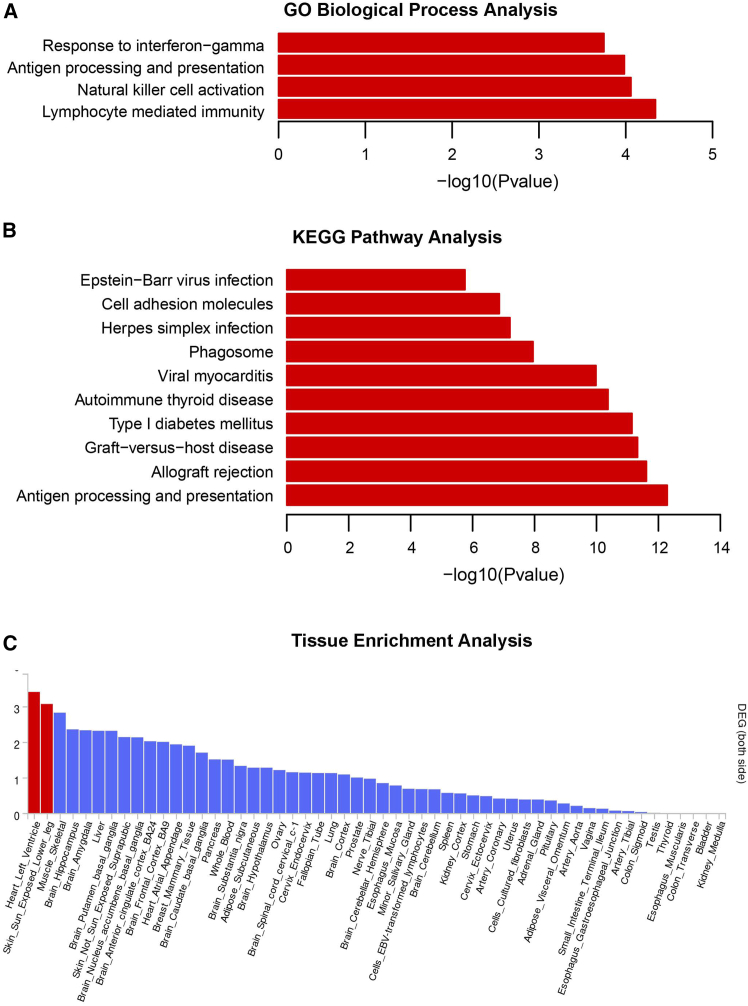
Pathway and tissue enrichment analysis of the 61 significant shared genes between immune cells and cancers (A) The significant findings of the GO biological process analysis after Benjamin-Hochberg correction. (B) The significant findings of the Kyoto Encyclopedia of Genes and Genomes (KEGG) pathway analysis after Benjamin-Hochberg correction. (C) Gene expression enrichment in all 54 tissues in the GTEx project. Red bar represents significant tissue enrichment after Benjamin-Hochberg correction.

To gain insights into the biological pathways and tissue enrichment associated with these 61 shared genes, we conducted a comprehensive series of analyses, as illustrated in Figure 4, which yielded significant findings. Specifically, our GO biological process analysis revealed enrichment in lymphocyte mediated immunity, natural killer cell activation, antigen processing and presentation, and response to interferon-gamma (FDR<0.03). Additionally, KEGG pathway analysis identified ten significant enrichments, including antigen processing and presentation, allograft rejection, graft-versus-host disease, type 1 diabetes mellitus, autoimmune thyroid disease, viral myocarditis, phagosome, herpes simplex infection, cell adhesion molecules, and Epstein-Barr virus infection (FDR<5.75 × 10^−5^). Furthermore, GTEx tissue enrichment analysis highlighted the significant enrichment of these shared genes in tissues, including the heart left ventricle and skin sun-exposed lower leg.

## Discussion

To the best of our knowledge, this study represents the most comprehensive analysis to date to systematically investigate both vertical pleiotropy (the impact of immune cells on cancers) and horizontal pleiotropy among 731 immune cell phenotypes and 16 site-specific cancers. Specifically, our MR analysis identified 79 significant causal associations involving 74 immune cells and 14 specific cancer types. Furthermore, evidence of shared genetic underpinnings, including 61 shared genes revealed by MAGMA analysis, four GO biological processes, 10 KEGG pathways, and enrichment in two tissues, underscores the complex interplay between immune cell biology and cancer susceptibility. Notably, we identified both vertical and horizontal pleiotropy among multiple immune cells in relation to melanoma, lung cancer, breast cancer, cervical cancer, and prostate cancer. These findings deepen our understanding of the intricate relationships between immune cells and cancer, with important implications for cancer prevention.

The immune system plays a pivotal role in human melanoma, a malignancy known for its complexity and genomic diversity. Our MR analysis identified causal associations between genetically elevated levels of immune cells (such as B cells, Treg cells, Myeloid cells, and cDC) and a decreased risk of melanoma. Previous studies documented similar findings. Initial investigations in a murine model of melanoma have indicated that B cells may exert both pro- and anti-tumorigenic effects, which often depend on the *in vivo* model system. Notably, B cell activation may also be critical for tumor regression in melanoma.^19^ Concurrently, studies in murine tumors have suggested that Treg cells inhibit the immune responses to tumors, and depletion of these cells has been shown to enhance rejection in various murine tumor models, including melanoma.^20^ Furthermore, our gene-based analysis identified *PLA2G6* shared between BAFF-R B cells and melanoma. The *PLA2G6*-encoded protein may play a role in phospholipid remodeling, arachidonic acid release, leukotriene and prostaglandin synthesis, fas-mediated apoptosis, and transmembrane ion fluxes in glucose-stimulated B cells.^21^ Meanwhile, Wang et al. utilized multiple methodologies, including Oncomine and CCLE online database, immunohistochemistry, RT-qPCR, and western blotting analyses, to demonstrate that *PLA2G6* knockdown significantly suppressed cell proliferation and metastasis, while promoting apoptosis in melanoma.^22^ These findings underscore the complex interplay between immune cell phenotypes and melanoma development.

The pathogenesis of lung cancer is complex, involving intricate interactions among tumor cells, stromal fibroblasts, and immune cells. Our MR analysis identified causal relationships indicating that genetically elevated levels of immune cells, particularly five types of B cells and a specific monocyte type, were associated with an increased risk of lung cancer. This finding is supported by previous studies showing that proliferating B cells are present in approximately 35% of lung cancer.^23^ Moreover, tumor-infiltrating B lymphocytes are observed at all stages of lung cancer development, and their presence varies between stages and histological subtypes, suggesting that B cells play a key role in the progression of lung cancer.^24^ Furthermore, our gene-based analysis identified 30 genes shared between 75 immune cells and lung cancer, all within the HLA region. Perea et al. demonstrated a strong correlation between tumor tissue structure and HLA-I expression in lung cancer.^25^ Additionally, McGranahan et al. identified that HLA loss of heterozygosity affects 40% of non-small-cell lung cancer based on sequencing data and was associated with a high subclonal neoantigen burden, APOBEC (apolipoprotein B mRNA-editing enzyme, catalytic polypeptide-like) mediated mutations, upregulated cytolytic activity, and programmed death ligand 1 positivity.^26^ These findings collectively highlight the critical role of immune cells, particularly B cells and HLA-related genetic variations, in the progression and immune evasion mechanisms of lung cancer.

The hypothesis that the immune system plays a causal role in reproductive cancer etiology is well-supported by epidemiological, preclinical, and clinical research findings.^15^^,^^27^^,^^28^^,^^29^ Our MR analysis confirmed the causal associations between genetically elevated levels of immune cells and a reduced risk of breast cancer, as well as an increased risk of cervical cancer. Interestingly, previous research has emphasized the importance of lymphocytes—especially T cells, Treg cells, and TNBK—as well as their cytokine profiles in both primary and secondary prevention strategies for breast cancer.^27^ Concurrently, recent studies indicated that during the development of cervical cancer, key immune cells—B cells, total T cells, Treg cells, monocytes, neutrophils, and M2-like macrophages—were increasingly involved as the disease progresses from normal to cancerous states.^30^ Furthermore, our gene-based analysis revealed three genes, including *PLA2G6*, *FAM83F*, and *ADSL,* that were shared between immune cells (primarily B cells) and breast cancer. Of these, *FAM83F*, an oncogene implicated in signaling pathways, showed significant associations with immune infiltration subtypes and adversely affects survival outcomes in breast cancer, highlighting its potential as a therapeutic target.^31^^,^^32^ *ADSL*, essential for *de novo* purine synthesis, was also noted as a substrate for EglN2 hydroxylase in triple-negative breast cancer.^33^^,^^34^ Additionally, *HLA-DRB1* and *HLA-DRA* were associated with 59 immune cells and cervical cancer. *HLA-DRB1*, which pairs with *HLA-DRA* to form a critical antigen-binding heterodimer, was associated with improved disease-free and disease-specific survival in cervical adenocarcinoma.^35^ Moreover, a meta-analysis also highlighted associations between multiple *HLA-DRB1* alleles and cervical cancer across various populations.^36^ In addition, although only six immune cells were causally associated with prostate cancer, 91% (56 out of 61) of these genes identified were associated with both prostate cancer and 103 immune cells in our gene-based analysis. A recent study suggested that both adaptive and innate immune cells play roles in the initiation, progression, metastasis, and treatment of prostate cancer.^29^ Meanwhile, multiple pieces of evidence suggested that prostate tumor-infiltrating immune cells can play a dichotomous role in promoting or inhibiting prostate carcinogenesis, depending on factors such as disease stage, inflammation type, and tumor microenvironment.^37^

Our study has several major strengths. To our knowledge, this is the first study to comprehensively evaluate both the vertical and horizontal relationships between systemic immune cells and site-specific cancers. This study covered the broadest range of immune cells and cancer types, providing a thorough assessment of the association between immune profiles and cancer risk. Furthermore, a comprehensive MR design based on independent significant loci and sensitivity analyses, as well as full summary data, is instrumental in ensuring the robustness of results. Through these stringent approaches, several potential false positives among previously reported causal relationships were identified by our findings. For instance, a previous study suggested that an elevated level of CD14^−^CD16+monocyte might be a protective factor against lung cancer,^34^ however, our findings did not support this. Although we observed a significant association using the IVW method (OR = 0.92, *p* = 3.87 × 10^−5^) and this was consistent across the MR-PRESSO, MR-Egger regression, and weighted median, however, MR-CAUSE yielded a null association.

Our findings hold significant implications for both public health and clinical practice. First, our study included the largest number of immune phenotypes and cancer types, providing valuable insights into potential mechanisms linking immune phenotypes and various cancers. Second, the identification of causal associations between specific immune cell phenotypes and various cancers highlights the potential for immune-based biomarkers to guide early detection and personalized prevention strategies. For example, BAFF-R expression on B cell subtypes associated with decreased melanoma risk, and CD27 on B cells linked to increased lung cancer risk, could potentially be integrated into risk stratification models to identify individuals who would benefit from enhanced screening or preventive interventions. Third, the extensive overlap of genes, particularly those located within the HLA region on chromosome 6 and associated with various cancers, underscores the potential for developing targeted immunotherapies and precision medicine approaches. The 61 shared genes we identified, enriched in antigen processing and lymphocyte-mediated immunity pathways, represent candidate targets for drug development and repurposing opportunities. Additionally, the shared genetic underpinnings across different cancers (e.g., genes associated with prostate cancer also linked to breast, cervical, and lung cancers) underscore the need for a holistic approach to cancer research, potentially leading to more effective cross-cancer therapies and improved patient outcomes. However, translating these insights into clinical practice requires validation in prospective cohorts with measured immune phenotypes and cancer outcomes, mechanistic studies elucidating how specific immune markers influence carcinogenesis, biomarker-driven clinical trials, and extension to diverse populations. This integrative approach not only enhances our understanding of cancer immunology but also offers promising avenues for improving diagnostic and therapeutic interventions across multiple cancer types.

### Limitations of the study

Our study has certain limitations. Firstly, GWAS studies on immune cells are currently limited to European populations, which may constrain the generalizability of our findings. Considering the genetic diversity across racial and ethnic groups, the genetic architecture and effect sizes of the immune-cancer relationships we identified may vary in non-European populations. Future studies incorporating multi-ancestry GWAS data will be essential to validate these associations and ensure their applicability across diverse populations. Secondly, we must note that both MR analysis and gene-based analysis are based on genome-wide summary-level data and therefore cannot substitute for direct clinical or experimental validation. Future research should integrate clinical samples with experimental approaches, such as single-cell transcriptomics, to further elucidate the underlying biological mechanisms. Thirdly, in our MR analysis, we did not perform multiple corrections for significance. This limitation is compounded by the relatively small sample size of the immune cell GWAS (up to 3,757 participants), which may have reduced statistical power. Consequently, only two associations passed the multiple testing correction based on the Benjamini-Hochberg method (FDR <0.05). Finally, the selection and specificity of genetic instruments for immune-cell traits are constrained by the current availability of GWAS data. Many immune-cell subsets are highly correlated and lack strong instruments, which may introduce weak-instrument bias and limit the interpretability of associations for closely related cell types.

## Resource availability

### Lead contact

Further information and requests for resources should be directed to and will be fulfilled by the lead contact, Xia Jiang (xia.jiang@ki.se).

### Materials availability

Only publicly available data were utilized in this study. No new unique reagents or biological materials were generated.

### Data and code availability

#### Data

GWAS summary statistics analyzed in this study are publicly available. Detailed sources and processing methods are provided in the key resources table.

#### Code

Additional supporting information and data are available from the lead contact upon reasonable request.

#### Other

All software tools used in this study—including Plink, the TwoSampleMR package, MR-PRESSO, MR CAUSE, and MAGMA—are publicly available and are documented in the Software and Algorithms section.

## STAR★Methods

### Key resources table

**Table:** 

REAGENT or RESOURCE	SOURCE	IDENTIFIER
**Deposited data**		

GWAS summary statistics for immune cells	Orrù et al.^38^	https://www.ebi.ac.uk/gwas/studies/GCST90001391 -https://www.ebi.ac.uk/gwas/studies/GCST90002121
GWAS summary statistics for bladder cancer	Sara R Rashkin et al.^39^	https://www.ebi.ac.uk/gwas/studies/GCST90011817
GWAS summary statistics for breast cancer	Zhang H et al.^40^	https://bcac.ccge.medschl.cam.ac.uk/bcacdata/oncoarray/oncoarray-and-combined-summary-result/gwas-summary-associations-breast-cancer-risk-2020/
GWAS summary statistics for cervical cancer	Lyon MS et al.^41^	https://gwas.mrcieu.ac.uk/datasets/ukb-b-8777/
GWAS summary statistics for colorectal cancer	Finngen R9	https://storage.googleapis.com/finngen-public-data-r9/summary_stats/finngen_R9_C3_COLORECTAL_EXALLC.gz
GWAS summary statistics for corpus uteri cancer	Finngen R9	https://storage.googleapis.com/finngen-public-data-r9/summary_stats/finngen_R9_C3_CORPUS_UTERI_EXALLC.gz
GWAS summary statistics for endometrial cancer	O'Mara TA et al.^42^	https://www.ebi.ac.uk/gwas/studies/GCST006464
GWAS summary statistics for kidney cancer	Laskar RS et al.^43^	https://www.ebi.ac.uk/gwas/studies/GCST008225, https://www.ebi.ac.uk/gwas/studies/GCST008226
GWAS summary statistics for liver cancer	Trépo E et al.^44^	https://www.ebi.ac.uk/gwas/studies/GCST90092003
GWAS summary statistics for lung cancer	James D McKay et al.^45^	https://www.ebi.ac.uk/gwas/studies/GCST004748
GWAS summary statistics for melanoma	Sara R Rashkin et al.^39^	https://www.ebi.ac.uk/gwas/studies/GCST90011809
GWAS summary statistics for oral cavity cancer	Lesseur C et al.^46^	https://www.ebi.ac.uk/gwas/studies/GCST012237
GWAS summary statistics for ovarian cancer	Phelan CM et al.^47^	https://www.ebi.ac.uk/gwas/studies/GCST004415
GWAS summary statistics for pancreatic cancer	Evangelina López de Maturana et al.^48^	https://www.ebi.ac.uk/gwas/studies/GCST90011858
GWAS summary statistics for prostate cancer	Anqi Wang et al.^49^	https://www.ebi.ac.uk/gwas/studies/GCST90274714
GWAS summary statistics for stomach cancer	Finngen R9	https://storage.googleapis.com/finngen-public-data-r9/summary_stats/finngen_R9_C3_STOMACH_EXALLC.gz
GWAS summary statistics for thyroid cancer	Finngen R9	https://storage.googleapis.com/finngen-public-data-r9/summary_stats/finngen_R9_C3_THYROID_GLAND_EXALLC.gz

**Software and algorithms**		

PLINK v2.0		https://www.cog-genomics.org/plink/2.0/
R v4.1.0		https://www.r-project.org/
TwoSampleMR v0.5.6	Hemani et al.^50^	https://www.mrbase.org/
MR PRESSO v1.0	Hartwig et al.^51^	https://github.com/rondolab/MR-PRESSO
MR CAUSE v1.2.0	Morrison et al.^52^	https://github.com/jean997/cause
MAGMA v1.10	de Leeuw et al.^53^	https://cncr.nl/research/magma/
WebGestalt	Liao et al.^54^	https://2019.webgestalt.org/
FUMA	Watanabe et al.^55^	https://fuma.ctglab.nl/home

### Experimental model and study participant details

The most comprehensive GWAS on immune cells was conducted by Orrù et al., involving 3,757 European individuals with no overlap among cohorts.^38^ This study analyzed 731 immune cells, categorized into absolute cell counts (n = 118), relative cell counts (n = 192), median fluorescence intensities (n = 389), and morphological parameters (n = 32). Specifically, absolute and relative cell counts, as well as median fluorescence intensities, included myeloid cells, B cells, mature stages of T cells, monocytes, TBNK (T cells, B cells, natural killer cells), CDCs, and Treg panels, while the morphological parameters category consisted of CDCs and TBNK panels. Details on the methodologies of this study can be found in the published research. Summary statistics data were accessed from the GWAS catalog (GCST90001391 to GCST90002121).

GWAS summary statistics for site-specific cancers were obtained from publicly available databases. Each study focused on individuals of European ancestry and included data on 16 cancers, totaling 364,597 cases and up to 2,974,017 controls. A summary of the number of cases and controls for each cancer, along with the data source, is provided in Table S5. Details on statistical analyses, imputation methods, and quality control procedures can be found in the respective original publications.

The summary statistics for immune cells and cancers were non-overlapping, and furthermore, ethical approval and participant consent were obtained for all original studies from which the data were derived.

### Method details

#### Mendelian randomization analysis

We conducted two-sample MR analyses to investigate the potential causal relationships between 731 immune cells and the risk of 16 site-specific cancers. The quality assessment of the MR analysis showed that our study adhered to the guidelines outlined in the recently released STROBE-MR statement, summarized in Data S2.^56^ Our IV selection strategy was designed to balance statistical power with the validity of causal inferences. Specifically, we adopted a genome-wide significance threshold of *P* < 1×10^-5^ and a linkage disequilibrium threshold of *r*^2^ < 0.001 within a 1000 kb window. This relaxed *P*-value threshold has been widely used in MR analyses of immune traits^38^^,^^57^^,^^58^ as it captures genetic variants with modest but reliable associations while providing a sufficient number of instruments for comprehensive sensitivity analyses. Immune cell traits with less than four IVs were excluded to ensure an adequate number of instruments for all MR methods (in particular for sensitivity analysis). The F-statistic threshold of 10 corresponds to instruments explaining >1% of exposure variance, effectively mitigating weak instrument bias that can lead to substantial type I error inflation in two-sample MR.^59^^,^^60^ Finally, immune cells selected for causal inference with site-specific cancer, along with details on their IVs and F-statistics, are shown in Data S3.

MR utilizes genetic variants as proxies for exposure, requiring that IVs satisfy three key assumptions: (i) the genetic variants used as IVs are directly associated with the exposure; (ii) the genetic variants are not associated with potential confounders; (iii) and the genetic variants exclusively affect outcome through the exposure, without involvement in any alternative causal pathways. In compliance with all assumptions being met, we employed the inverse variance weighted (IVW) method as our primary approach^61^ to estimate causal effects, assuming the validity of all instrumental variables (IVs) for maximum statistical power.

To ensure the robustness of our findings, we conducted additional sensitivity analyses, including (i) MR Egger regression^62^ to detect and adjust for bias arising from directional pleiotropy, (ii) the weighted median approach^63^ to provide a consistent causal estimation even when more than 50% of IVs may be invalid, and (iii) MR PRESSO (MR-Pleiotropy Residual Sum and Outlier)^51^ to identify and correct for horizontal pleiotropy, recalculating causal effects after removing identified outliers. Consistent findings across these methods suggest a credible causal estimate. Therefore, we considered an estimated causal relationship as suggestive if the P-value was < 0.05 in IVW, with consistent effect size direction across all sensitivity methods. To further assess the robustness of suggestive associations, we performed leave-one-out analyses^50^ using multiplicative random effects IVW method to evaluate the impact of outlier and pleiotropic SNPs on causal estimates.

To establish robust causal effects for these suggestive associations that passed the leave-one-out analysis, we employed the Causal Analysis Using Summary Effect Estimates (CAUSE) method.^52^ Integrating information from genome-wide SNPs, CAUSE has several advantages over the conventional MR approaches: it accounts for both uncorrelated and correlated horizontal pleiotropy, improves statistical power, and reduces the likelihood of false positives. Under a Bayesian framework, CAUSE incorporates a parameter (q value) representing the proportion of variants likely exhibiting correlated horizontal pleiotropy. It provides posterior distribution estimates under two models: the sharing model, which considers only horizontal pleiotropic effects, and the causal model, which accommodates both horizontal pleiotropy and causality. A one-sided *P*_causal vs. sharing_ is generated to evaluate whether the sharing model is at least as effective as the causal model in fitting the data, with a rejection of the null hypothesis (*P*_causal vs. sharing_ < 0.05) indicating that the data are more likely explained by causal effects. Therefore, we defined a significant causal relationship as *P*_causal vs. sharing_ < 0.05 in CAUSE.

#### Gene-based analysis

To investigate horizontal pleiotropy, specifically genetic variants that have independent effects on both immune cells and cancers, we conducted a gene-based association analysis using Multi-marker Analysis of GenoMic Annotation (MAGMA),^53^ which aggregates genetic variants to the gene level and assesses their collective association with the phenotype. The MAGMA analysis involves two primary steps: firstly, an annotation step to map SNPs to genes utilizing the 19,427 protein-coding genes in NCBI build 37; secondly, a gene-level analysis step to calculate p-values using the 1KGP Phase 3 panel of European ancestry. In our analysis, we initially performed MAGMA analyses for immune cell phenotypes and cancers respectively, to identify significant genes with a threshold of *P* < 0.05/19,427 associated with each phenotype. Subsequently, to improve robustness and minimize false positives among the 731 immune traits, we further required each gene to reach significance in at least 10 immune cell phenotypes. This empirical criterion prioritized genes with consistent associations across multiple immune traits, suggesting broad immune regulatory relevance. Finally, we determined the intersection of these genes with the significant genes associated with site-specific cancer.

#### Pathway and tissue enrichment analysis

To explore the shared biological pathways and tissue enrichments between immune cells and 16 site-specific cancers, we conducted functional enrichment using multiple resources for genes identified through MAGMA analysis. We applied the WebGestalt tool^54^ to assess the enrichment of all the shared genes in Gene Ontology (GO) biological processes and Kyoto Encyclopedia of Genes and Genomes (KEGG) pathways. We also performed gene set tissue enrichment analysis using the GENE2FUNC process in functional mapping and annotation of genome-wide association studies (FUMA)^55^ with 54 tissue types available from GTEx (version 8). The Benjamin-Hochberg procedure was used to correct for multiple testing.

### Quantification and statistical analysis

All statistical analyses and figure generation were performed using R software (version 4.2.1). Specific analyses were conducted with the following packages and tools: MR analyses were implemented using the TwoSampleMR (version 0.5.6), MR-PRESSO (version 1.0), and cause (version 1.2.0) packages; Gene-based association analysis was performed with MAGMA (version 1.10); Pathway and tissue enrichment analyses were carried out using the online tool WebGestalt and FUMA, respectively.

## Acknowledgments

This study was supported by the 10.13039/501100012166National Key R&D Program of China (2022YFC3600600), the 10.13039/501100001809National Natural Science Foundation of China (U22A20359, 81874283, 81673255), the Science Fund for Creative Research Groups of science and Technology Bureau of Sichuan Province (2024NSFTD0030), the 10.13039/501100018549Recruitment Program for Young Professionals of China, the Science Fund for Creative Research Groups of Science and Technology Bureau of Sichuan Province, the Promotion Plan for Basic Medical Sciences and the Development Plan for Cutting-Edge Disciplines, Sichuan University, and other Projects from West China School of Public Health and West China Fourth Hospital, Sichuan University.

## Author contributions

X.J. and B.Z. designed the study. M.T. performed the data analyses. Y.W., Y.Z., R.X., B.L., F.M., D.Z., and L.C. checked the results. Y.X., Y.L., Q.Z., M.F., and Y.L. drew figures. M.T. drafted the article. H.C., X.W., and X.J. revised the article, and all authors read and approved the final article.

## Declaration of interests

All authors declared that there are no conflicts of interest in relation to the subject of this study.

## Supplemental information

Document S1. Figure S1 and Data S2

Table S1. Leave-one-out analyses for the effect of 507 significant associations between immune cells and cancer were identified by Mendelian randomization based on independent significant loci for immune cell

Table S2. 501 Associations between immune cells and cancers by MR-CAUSE

Table S3. 79 significant associations between immune cells and cancers were identified by five Mendelian randomization methods

Table S4. Gene-based association between immune cells and cancers by MAGMA

Table S5. Summary statistics data sources

Data S1. Summary of Mendelian Randomization Analysis Results

Data S3. Summary of Immune traits and their instrumental variables and F-statistics
