# Supplementary material for: Association between immune cells and multiple cancers: Insights from Mendelian randomization and gene-based analysis
Source: iScience. 2026 Jan 2;29(2):114618. doi: 10.1016/j.isci.2025.114618 (PMC12857361; doi:10.1016/j.isci.2025.114618)
Supplement: Document S1. Figure S1 and Data S2 [file mmc1.pdf]

iScience, Volume *29*

Supplemental information

Association between immune cells and multiple cancers: Insights from Mendelian randomization and gene-based analysis

Mingshuang Tang, Huijie Cui, Xueyao Wu, Yutong Wang, Xunying Zhao, Rong Xiang, Jinyu Xiao, Lin Chen, Yanqiu Zou, Yunjie Liu, BoWen Lei, Xiaofeng Ma, Di Zhang, Mengyu Fan, Jiayuan Li, Xia Jiang, and Ben Zhang

Supplemental Information

Association between Immune Cells and Multiple Cancers: Insights from Mendelian Randomization and Gene-based Analysis

Mingshuang Tang, Huijie Cui, Xueyao Wu, Yutong Wang, Xunying Zhao, Rong Xiang, Jinyu Xiao, Lin Chen, Yanqiu Zou, Yunjie Liu, BoWen Lei, Xiaofeng Ma, Di Zhang, Mengyu Fan, Jiayuan, Li, Xia Jiang, Ben Zhang

1. Supplementary Figure

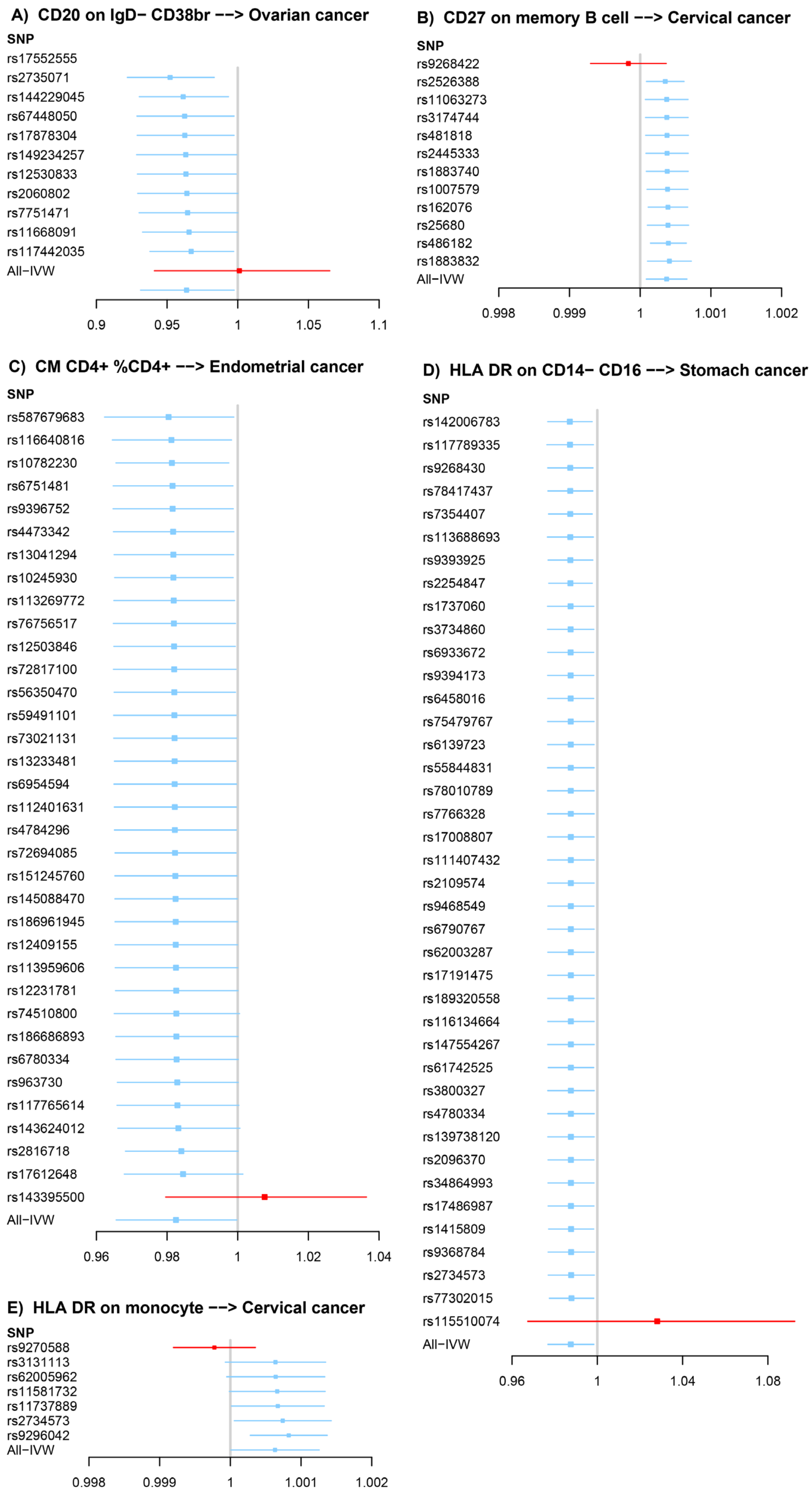

Figure S1. The results of Leave-one-out sensitivity analysis for five immune cell-cancer associations influenced by outliers. Panel A (CD20 on IgD- CD38br with ovarian cancer), Panel B (CD27 on memory B cell with cervical cancer), and Panel C (CM CD4+ %CD4+ with endometrial cancer), Panel D (HLA DR on CD14- CD16 with stomach cancer), and Panel E (HLA DR on monocyte with cervical cancer) show the estimates of causal associations. In each panel, dots and horizontal lines represent the MR estimates and their 95% confidence intervals after sequentially removing individual SNPs from the analysis.

2. Supplementary Table

Data S2. STROBE-MR checklist of recommended items to address in reports of Mendelian randomization studies.

Item No.	Section	Checklist item	Page No.	Relevant text from manuscript
1	TITLE and ABSTRACT	Indicate Mendelian randomization (MR) as the study's design in the title and/or the abstract if that is a main purpose of the study	1	Association Between Immune cells and Multiple Cancers: Insights from Mendelian Randomization and Gene-base Analysis
	INTRODUCTION			
2	Background	Explain the scientific background and rationale for the reported study. What is the exposure? Is a potential causal relationship between exposure and outcome plausible? Justify why MR is a helpful method to address the study question	4	Immune cells are essential in both the development and progression of cancer [1,2]. Traditional understanding posits that intact immune responses—such as immune surveillance or immunoediting—are essential for preventing and inhibiting cancer development [3,4]. Conversely, accumulating evidence has elucidated specific mechanisms of immune regulation in carcinogenesis, such as pro-tumorigenic inflammation, immune surveillance suppression mediated by TH17 and regulatory T cells (Tregs), TH1-mediated immunosuppression, and local and distant tumorigenesis regulated by

				microbiota via alterations in the inflammatory and metabolic pathways [5-8].
3	Objectives	State specific objectives clearly, including pre-specified causal hypotheses (if any). State that MR is a method that, under specific assumptions, intends to estimate causal effects	5	As phenotypic correlations derived from observational designs can be subject to bias, confounding, and reverse causality, Mendelian randomization (MR) analysis offers more robust insights into the causal associations between risk factors and disease, most closely resembling a randomized controlled trial (RCT) [13]. Published MR analyses on the relationship between immune cells and cancer risk have covered several cancers including breast [14,15], ovarian [15], endometrial [15], lung [16,17], and gastrointestinal tract cancers [18]. Additionally, inconsistencies in MR methods across these studies have made it challenging to compare the robustness of associations. Therefore, a comprehensive study to assess the relationship between various immune cells and multiple site-specific cancers using a systematic approach is needed.<br>To sum up, in this study we aimed to investigate the relationship between 731 genetically predicted immune cell

				phenotypes and 16 site-specific cancers with available genome-wide association studies (GWAS) data, by conducting MR analysis, followed by gene-based analysis, as well as pathway and tissue enrichment analyses.
	METHODS			
4	Study design and data sources	Present key elements of the study design early in the article. Consider including a table listing sources of data for all phases of the study. For each data source contributing to the analysis, describe the following:	NA	
	a)	*Setting: Describe the study design and the underlying population, if possible. Describe the setting, locations, and relevant dates, including periods of recruitment, exposure, follow-up, and data collection, when available.*	*7*<br>*6*<br>*6*	We conducted two-sample MR analyses to investigate the potential causal relationships between 731 immune cells and the risk of 16 site-specific cancers. The quality assessment of the MR analysis showed that our study adhered to the guidelines outlined in the recently released STROBE-MR statement, summarized in **Data S2** [39].<br>Exposure: The most comprehensive GWAS on immune cells was conducted by Orrù et al., involving 3,757 European individuals with no overlap among cohorts 38. This study analyzed 731 immune cells, categorized into absolute cell counts (n = 118), relative cell counts (n = 192), median fluorescence

			intensities (n = 389), and morphological parameters (n = 32). Specifically, absolute and relative cell counts, as well as median fluorescence intensities, included myeloid cells, B cells, mature stages of T cells, monocytes, TBNK (T cells, B cells, natural killer cells), CDCs, and Treg panels, while the morphological parameters category consisted of CDCs and TBNK panels. Details on the methodologies of this study can be found in the published research. Summary statistics data were accessed from the GWAS catalog (GCST90001391 to GCST90002121).<br>Outcome: The most comprehensive GWAS on immune cells was conducted by Orrù et al., involving 3,757 European individuals with no overlap among cohorts 38. This study analyzed 731 immune cells, categorized into absolute cell counts (n = 118), relative cell counts (n = 192), median fluorescence intensities (n = 389), and morphological parameters (n = 32).
b)	Participants: Give the eligibility criteria, and the sources and methods of selection of participants. Report the sample size, and whether any power or sample size calculations were carried out prior to the main analysis	6	Exposure: The most comprehensive genome-wide association studies (GWAS) on immune cells was conducted by Orrù et al., involving 3,757 European individuals with no overlap

			among cohorts [17]. This study analyzed 731 immune cells, categorized into absolute cell counts (n = 118), relative cell counts (n = 192), median fluorescence intensities (n = 389), and morphological parameters (n = 32).<br>Outcome: The most comprehensive GWAS on immune cells was conducted by Orrù et al., involving 3,757 European individuals with no overlap among cohorts 38. This study analyzed 731 immune cells, categorized into absolute cell counts (n = 118), relative cell counts (n = 192), median fluorescence intensities (n = 389), and morphological parameters (n = 32).
c)	Describe measurement, quality control and selection of genetic variants	7	Our IV selection strategy was designed to balance statistical power with the validity of causal inferences. Specifically, we adopted a genome-wide significance threshold of P < 1×10-5 and a linkage disequilibrium threshold of $r2$ < 0.001 within a 1000 kb window. This relaxed P-value threshold has been widely used in MR analyses of immune traits 38,40,41 as it captures genetic variants with modest but reliable associations while providing a sufficient number of instruments for comprehensive sensitivity analyses.

				Immune cell traits with less than four IVs were excluded to ensure an adequate number of instruments for all MR methods (in particular for sensitivity analysis). The F-statistic threshold of 10 corresponds to instruments explaining >1% of exposure variance, effectively mitigating weak instrument bias that can lead to substantial type I error inflation in two-sample MR 42,43. Finally, immune cells selected for causal inference with site-specific cancer, along with details on their IVs and F-statistics, are shown in Data S3.
	d)	For each exposure, outcome, and other relevant variables, describe methods of assessment and diagnostic criteria for diseases		
	e)	*Provide details of ethics committee approval and participant informed consent, if relevant*	*7*	The summary statistics of immune cells and cancers were not overlapping, and all participants provided informed consent in all the corresponding original studies.
5	Assumptions	Explicitly state the three core IV assumptions for the main analysis (relevance, independence and exclusion restriction) as well assumptions for any additional or sensitivity analysis	8	MR utilizes genetic variants as proxies for exposure, requiring that IVs satisfy three key assumptions: (i) the genetic variants used as IVs are directly associated with the exposure; (ii) the genetic variants are not associated with potential confounders; (iii) and the genetic variants exclusively affect outcome

				through the exposure, without involvement in any alternative causal pathways.
6	Statistical methods: main analysis	Describe statistical methods and statistics used	8	In compliance with all assumptions being met, we employed the inverse variance weighted (IVW) method as our primary approach 44 to estimate causal effects, assuming the validity of all instrumental variables (IVs) for maximum statistical power.
	a)	Describe how quantitative variables were handled in the analyses (i.e., scale, units, model)	NA	
	b)	Describe how genetic variants were handled in the analyses and, if applicable, how their weights were selected	NA	
	c)	Describe the MR estimator (e.g. two-stage least squares, Wald ratio) and related statistics. Detail the included covariates and, in case of two-sample MR, whether the same covariate set was used for adjustment in the two samples	8	IVW
	d)	Explain how missing data were addressed	NA	
	e)	If applicable, indicate how multiple testing was addressed	NA	
7	Assessment of assumptions	Describe any methods or prior knowledge used to assess the assumptions or justify their validity	8	Our IV selection strategy was designed to balance statistical power with the validity of causal inferences. Specifically, we adopted a genome-wide significance threshold of P < 1×10-5 and a linkage disequilibrium threshold of $r2$ < 0.001 within a 1000 kb window. This relaxed P-value threshold has

				been widely used in MR analyses of immune traits 38,40,41 as it captures genetic variants with modest but reliable associations while providing a sufficient number of instruments for comprehensive sensitivity analyses.
8	Sensitivity analyses and additional analyses	Describe any sensitivity analyses or additional analyses performed (e.g. comparison of effect estimates from different approaches, independent replication, bias analytic techniques, validation of instruments, simulations)	8-9	To ensure the robustness of our findings, we conducted additional sensitivity analyses, including (i) MR Egger regression 45 to detect and adjust for bias arising from directional pleiotropy, (ii) the weighted median approach 46 to provide a consistent causal estimation even when more than 50% of IVs may be invalid, and (iii) MR PRESSO (MR-Pleiotropy Residual Sum and Outlier) 47 to identify and correct for horizontal pleiotropy, recalculating causal effects after removing identified outliers. Consistent findings across these methods suggest a credible causal estimate. Therefore, we considered an estimated causal relationship as suggestive if the P-value was < 0.05 in IVW, with consistent effect size direction across all sensitivity methods. To further assess the robustness of suggestive associations, we performed leave-one-out analyses 48 using multiplicative random effects IVW method to

				evaluate the impact of outlier and pleiotropic SNPs on causal estimates.
9	Software and pre-registration			
	a)	Name statistical software and package(s), including version and settings used		
	b)	State whether the study protocol and details were pre-registered (as well as when and where)		No.
	RESULTS			
10	Descriptive data			
	a)	Report the numbers of individuals at each stage of included studies and reasons for exclusion. Consider use of a flow diagram	11	In MR analyses, we identified 506 suggestive causal associations using independent significant loci associated with immune cells, where the P-value was < 0.05 in IVW, and consistent effect size direction across all complementary methods, including MR-Egger regression, weighted median, and MR-PRESSO (Data S1). However, the leave-one-out analysis results revealed that the causal estimates of five associations were affected by outliers (see Table S1, Figure S1). Consequently, 501 associations were included in the MR CAUSE analysis (using genome-wide summary results associated with immune cells) Table S2), of which 79 were found to be statistically significant (Figure 2, Table S3).

	b)	Report summary statistics for phenotypic exposure(s), outcome(s), and other relevant variables (e.g. means, SDs, proportions)		Table S5
	c)	If the data sources include meta-analyses of previous studies, provide the assessments of heterogeneity across these studies	NA	
	d)	For two-sample MR:<br>i. Provide justification of the similarity of the genetic variant-exposure associations between the exposure and outcome samples<br>ii. Provide information on the number of individuals who overlap between the exposure and outcome studies	NA	
11	Main results			
	a)	Report the associations between genetic variant and exposure, and between genetic variant and outcome, preferably on an interpretable scale		Data S1
	b)	Report MR estimates of the relationship between exposure and outcome, and the measures of uncertainty from the MR analysis, on an interpretable scale, such as odds ratio or relative risk per SD difference		Data S3;<br>Table S2;<br>Table S3.
	c)	If relevant, consider translating estimates of relative risk into absolute risk for a meaningful time period	NA	
	d)	Consider plots to visualize results (e.g. forest plot, scatterplot of associations between genetic variants and outcome versus between genetic variants and exposure)		Figure 2
12	Assessment of assumptions			
	a)	Report the assessment of the validity of the assumptions	11	we identified 506 suggestive causal associations using independent significant loci associated with immune cells, where the P-value was < 0.05 in IVW, and consistent effect

				size direction across all complementary methods, including MR-Egger regression, weighted median, and MR-PRESSO (Data S1). However, the leave-one-out analysis results revealed that the causal estimates of five associations were affected by outliers (see Table S1, Figure S1). Consequently, 501 associations were included in the MR CAUSE analysis (using genome-wide summary results associated with immune cells) Table S2), of which 79 were found to be statistically significant (Figure 2, Table S3).
	b)	Report any additional statistics (e.g., assessments of heterogeneity across genetic variants, such as I2, Q statistic or E-value)	NA	
13	Sensitivity analyses and additional analyses			
	a)	Report any sensitivity analyses to assess the robustness of the main results to violations of the assumptions		Data S3;<br>Table S1;<br>Table S2;<br>Table S3.
	b)	Report results from other sensitivity analyses or additional analyses		Data S3;<br>Table S1;<br>Table S2;<br>Table S3.

	c)	Report any assessment of direction of causal relationship (e.g., bidirectional MR)	NA	
	d)	When relevant, report and compare with estimates from non-MR analyses	NA	
	e)	Consider additional plots to visualize results (e.g., leave-one-out analyses)	NA	
	DISCUSSION			
14	Key results	Summarize key results with reference to study objectives	14	To the best of our knowledge, this study represents the most comprehensive analysis to date to systematically investigate both vertical pleiotropy (the impact of immune cells on cancers) and horizontal pleiotropy among 731 immune cell phenotypes and 16 site-specific cancers. Specifically, our MR analysis identified 79 significant causal associations involving 74 immune cells and 14 specific cancer types.
15	Limitations	Discuss limitations of the study, taking into account the validity of the IV assumptions, other sources of potential bias, and imprecision. Discuss both direction and magnitude of any potential bias and any efforts to address them	18	Firstly, GWAS studies on immune cells are currently limited to European populations, which may constrain the generalizability of our findings. Considering the genetic diversity across racial and ethnic groups, the genetic architecture and effect sizes of the immune-cancer relationships we identified may vary in non-European populations. Future studies incorporating multi-ancestry GWAS data will be essential to validate these associations and

				ensure their applicability across diverse populations. Secondly, we must note that both MR analysis and gene-based analysis are based on genome-wide summary-level data and therefore cannot substitute for direct clinical or experimental validation. Future research should integrate clinical samples with experimental approaches, such as single-cell transcriptomics, to further elucidate the underlying biological mechanisms. Thirdly, in our MR analysis, we did not perform multiple corrections for significance. This limitation is compounded by the relatively small sample size of the immune cell GWAS (up to 3,757 participants), which may have reduced statistical power. Consequently, only two associations passed the multiple testing correction based on the Benjamini-Hochberg method (FDR < 0.05).
16	Interpretation			
	a)	Meaning: Give a cautious overall interpretation of results in the context of their limitations and in comparison with other studies	18	To our knowledge, this is the first study to comprehensively evaluate both the vertical and horizontal relationships between systemic immune cells and site-specific cancers. This study covered the broadest range of immune cells and cancer types, providing a thorough

			assessment of the association between immune profiles and cancer risk. Furthermore, a comprehensive MR design based on independent significant loci and sensitivity analyses, as well as full summary data, is instrumental in ensuring the robustness of results. Through these stringent approaches, several potential false positives among previously reported causal relationships were identified by our findings. For instance, a previous study suggested that elevated level of CD14 － CD16 ＋ monocyte might be a protective factor against lung cancer [46], however, our findings did not support this. Although we observed a significant association using the IVW method (OR = 0.92, P = 3.87×10-5) and this was consistent across the MR-PRESSO, MR-Egger regression, and weighted median, however, MR-CAUSE yielded null association.
b)	Mechanism: Discuss underlying biological mechanisms that could drive a potential causal relationship between the investigated exposure and the outcome, and whether the gene-environment equivalence assumption is reasonable. Use causal language carefully, clarifying that IV estimates may provide causal effects only under certain assumptions	19	First, our study included the largest number of immune phenotypes and cancer types, providing valuable insights into potential mechanisms linking immune phenotypes and various cancers. Second, the identification of causal associations between specific immune

			cell phenotypes and various cancers highlight the potential for immune-based biomarkers to guide early detection and personalized prevention strategies. For example, BAFF-R expression on B cell subtypes associated with decreased melanoma risk, and CD27 on B cells linked to increased lung cancer risk, could potentially be integrated into risk stratification models to identify individuals who would benefit from enhanced screening or preventive interventions.
c)	Clinical relevance: Discuss whether the results have clinical or public policy relevance, and to what extent they inform effect sizes of possible interventions	19	our study revealed significant instances of both vertical and horizontal pleiotropy between immune cell phenotypes and several cancers, including melanoma, lung cancer, breast cancer, cervical cancer, and prostate cancer. These pleiotropic effects highlight the interconnectedness of immune cell functions and their potential impact on specific cancers. Our findings suggest potential biological mechanisms that link immune cells to cancer development, progression, and prognosis, highlighting the complexity of the immune system's role in cancer.

17	Generalizability	Discuss the generalizability of the study results (a) to other populations, (b) across other exposure periods/timings, and (c) across other levels of exposure	NA	
	OTHER INFORMATION			
18	Funding	Describe sources of funding and the role of funders in the present study and, if applicable, sources of funding for the databases and original study or studies on which the present study is based		This study was supported by the National Key R&D Program of China (2022YFC3600600), the National Natural Science Foundation of China (U22A20359, 81874283, 81673255), the Science Fund for Creative Research Groups of science and Technology Bureau of Sichuan Province (2024NSFTD0030), the Recruitment Program for Young Professionals of China, the Science Fund for Creative Research Groups of Science and Technology Bureau of Sichuan Province, the Promotion Plan for Basic Medical Sciences and the Development Plan for Cutting-Edge Disciplines, Sichuan University, and other Projects from West China School of Public Health and West China Fourth Hospital, Sichuan University.
19	Data and data sharing	Provide the data used to perform all analyses or report where and how the data can be accessed and reference these sources in the article. Provide the		Table S5

		statistical code needed to reproduce the results in the article, or report whether the code is publicly accessible and if so, where		
20	Conflicts of Interest	All authors should declare all potential conflicts of interest	19	All authors declared that there are no conflicts of interest in relation to the subject of this study.
