# Supplementary material for: Association between immune cells and multiple cancers: Insights from Mendelian randomization and gene-based analysis
Source: iScience. 2026 Jan 2;29(2):114618. doi: 10.1016/j.isci.2025.114618 (PMC12857361; doi:10.1016/j.isci.2025.114618)
Supplement: Table S5. Summary statistics data sources [file mmc6.pdf]

Table S5. Summary statistics data sources

Trait	Resource	Sample size	Populatior	Reference	Download Site
**Immune cells**	GWAS Catalog	1,244 - 3,669	European	Orrù V,et al.Nat Genet.2020.PMID: 32929287.	https://www.ebi.ac.uk/gwas/studies/GCST90001391 - https://www.ebi.ac.uk/gwas/studies/GCST90002121
**Bladder cancer**	GWAS Catalog	case: 2,242, control: 410，350	European	Sara R Rashkin,et al.Nat Commun.2020.PMID:32887889	https://www.ebi.ac.uk/gwas/studies/GCST90011817
**Breast cancer**	Breast Cancer Association	case: 133,384, control: 113,789	European	Zhang H, et al. Nat Genet. 2020. PMID: 32424353.	https://bcac.ccge.medschl.cam.ac.uk/bcacdata/oncoarray/oncoarray-and-combined-summary-result/gwas-summary-associations-breast-cancer-risk-2020/
**Cervical cancer**	IEU OpenGWAS project	case: 1889, control: 461,044	European	Lyon MS, et al. Genome Biol. 2021. PMID: 33441155.	https://gwas.mrcieu.ac.uk/datasets/ukb-b-8777/
**Colorectal cancer**	Finngen	case: 6,509, control: 287,137	European	Finngen R9	https://storage.googleapis.com/finngen-public-data-r9/summary_stats/finngen_R9_C3_COLORECTAL_EXALLC.gz
**Corpus uteri cancer**	Finngen	case: 1,967, control: 167,189	European	Finngen R9	https://storage.googleapis.com/finngen-public-data-r9/summary_stats/finngen_R9_C3_CORPUS_UTERI_EXALLC.gz
**Endometrial cancer**	GWAS Catalog	case: 12,906, control: 108,979	European	O'Mara TA, et al. Nat Commun. 2018. PMID: 30093612.	https://www.ebi.ac.uk/gwas/studies/GCST006464
**Kidney cancer**	GWAS Catalog	case: 5219, control: 8011	European	Laskar RS, et al. Eur J Hum Genet. 2019. PMID: 31231134.	https://www.ebi.ac.uk/gwas/studies/GCST008225, https://www.ebi.ac.uk/gwas/studies/GCST008226
**Liver cancer**	GWAS Catalog	case: 775, control: 1332	European	Trépo E, et al. Lancet Oncol. 2022. PMID: 34902334.	https://www.ebi.ac.uk/gwas/studies/GCST90092003
**Lung cancer**	GWAS Catalog	case:29,266, control: 56,450	European	James D McKay, et al.Nat Genet.2017.PMID: 28604730.	https://www.ebi.ac.uk/gwas/studies/GCST004748
**Melanoma**	GWAS Catalog	case:6,777 , control: 410,350	European	Sara R Rashkin, et al.Nat Commun.2020.PMID:32887889.	https://www.ebi.ac.uk/gwas/studies/GCST90011809
**Oral cavity cancer**	GWAS Catalog	case:1,223, control: 2,928	European	Lesseur C, et al. Nat Genet. 2016. PMID: 27749845.	https://www.ebi.ac.uk/gwas/studies/GCST012237
**Ovarian cancer**	GWAS Catalog	case: 22,406, control: 40,941	European	Phelan CM, et al. Nat Genet. 2017. PMID: 28346442.	https://www.ebi.ac.uk/gwas/studies/GCST004415
**Pancreatic cancer**	GWAS Catalog	case: 1,317, control: 1,616	European	Evangelina López de Maturana.Genome Med.2021.PMID:335178	https://www.ebi.ac.uk/gwas/studies/GCST90011858
**Prostate cancer**	GWAS Catalog	case: 122,188, control: 604,640	European	Anqi Wang,et al.Nat Genet.2023.PMID: 37945903.	https://www.ebi.ac.uk/gwas/studies/GCST90274714
**Stomach cancer**	Finngen	case: 1,307, control: 287,137	European	Finngen R9	https://storage.googleapis.com/finngen-public-data-r9/summary_stats/finngen_R9_C3_STOMACH_EXALLC.gz
**Thyroid cancer**	Finngen	case: 1,783, control: 287,137	European	Finngen R9	https://storage.googleapis.com/finngen-public-data-r9/summary_stats/finngen_R9_C3_THYROID_GLAND_EXALLC.gz
